# Working on gender equality in the organization of the psychiatry association: can we do what we say?

**DOI:** 10.1192/j.eurpsy.2026.10271

**Published:** 2026-07-31

**Authors:** S. Erdogan Taycan

**Affiliations:** 1 Psychiatric Association; 2 Psychology, Istanbul Okan University, Istanbul, Türkiye

## Abstract

**Abstract:**

While increased awareness in today’s socioeconomic conditions has led to some positive progress in areas related to gender equality, we are still far from the ideal. Achieving gender equality, ensuring equal representation and rights for women in all settings and systems, should occur naturally; however, this is only partially possible through various regulations and their monitoring. Although professional organizations generally have a high level of awareness on these issues, internalized patriarchal codes can prevent our female colleagues from achieving adequate representation and having a voice in the process. Developing awareness and planning sustainable practices in this area is our responsibility and duty. I would like to introduce you to a series of initiatives we have implemented within the Psychiatric Association of Türkiye. In 2018, a policy document was prepared by a task force composed of knowledgeable colleagues in the field. A unit was established under the same name as this document on preventing sexual violence and supporting gender equality. In addition to its primary task of evaluating and supporting complainants in sexual abuse investigations where one of the parties is a psychiatrist, this unit also develops activities to support gender equality. Coordinating with other working units and task groups operating under our association, this unit recently prepared a Guide to Using Discrimination-Sensitive Language and Creating Content. We are working to promote the widespread use of this guide, which also includes gender equality issues, in all the association’s scientific activities. Through informational texts placed in congress’ bag, group work, and workshops at our association’s twice-yearly congresses, we aim to keep the theme of gender equality on the agenda in the theory and practice of our members. This year, during the week of March 8th, International Women’s Day, we are preparing a webinar where we will discuss the representation rates of women in all units and elected boards of our association, listen to suggestions and experiences from senior female colleagues in active roles, and discuss the expectations and challenges of young female colleagues. With the awareness of being part of and transforming the bigger picture, we need to discuss together the importance and impact of professional organizations taking an active role in the field of gender equality, starting from their own internal structures.

**Disclosure of Interest:**

None Declared

